# Artificial Intelligence in Predicting the Mode of Delivery: A Systematic Review

**DOI:** 10.7759/cureus.69115

**Published:** 2024-09-10

**Authors:** Kalliopi Michalitsi, Dimitra Metallinou, Athina Diamanti, Vasiliki E Georgakopoulou, Iraklis Kagkouras, Eleni Tsoukala, Antigoni Sarantaki

**Affiliations:** 1 Department of Midwifery, University of West Attica, Athens, GRC; 2 Department of Pathophysiology/Pulmonology, Laiko General Hospital, Athens, GRC; 3 Department of Surgery, Worcestershire Acute Hospital, Worcester, GBR; 4 Department of Obstetrics and Gynecology, IASO Maternity – Gynecology Hospital, Athens, GRC

**Keywords:** artificial intelligence, cesarian, machine learning, mode of delivery prediction, obstetrics, vaginal birth

## Abstract

The integration of artificial intelligence (AI) into obstetric care offers significant potential to enhance clinical decision-making and optimize maternal and neonatal outcomes. Traditional prediction methods for mode of delivery often rely on subjective clinical judgment and limited statistical models, which may not fully capture complex patient data. This systematic review aims to evaluate the current state of research on AI applications in predicting the mode of delivery, comparing the performance of AI models with traditional methods, and identifying gaps for future research. A comprehensive literature search was conducted across PubMed, Google Scholar, Web of Science, and Scopus databases, covering publications from January 2010 to July 2024. Inclusion criteria were studies employing AI techniques to predict the mode of delivery, published in peer-reviewed journals, and involving human subjects. Studies were assessed for quality using the Prediction Model Risk of Bias Assessment Tool (PROBAST), and data were synthesized narratively due to heterogeneity. In total, 18 studies met the inclusion criteria, employing various AI models such as logistic regression, random forest, gradient boosting, and neural networks. Sample sizes ranged from 40 to 94,480 participants across diverse geographic settings. AI models demonstrated high accuracy rates, often exceeding 90%, and strong predictive metrics (area under the curve (AUC) values from 0.745 to 0.932). Key predictors included maternal age, gravidity, parity, gestational age, labor induction type, and fetal weight. Notable models like the Adana System and Categorical Boosting (CatBoost, Yandex LLC, Moscow, Russia) highlighted the effectiveness of AI in enhancing prediction accuracy and supporting clinical decisions. AI models significantly outperform traditional statistical methods in predicting the mode of delivery, providing a robust tool for obstetric care. Future research should focus on standardizing data collection, improving model interpretability, addressing ethical concerns, and ensuring fairness in AI predictions to enhance clinical trust and application.

## Introduction and background

The advent of artificial intelligence (AI) has revolutionized various fields, including healthcare, where it is increasingly employed to enhance clinical decision-making and predict patient outcomes. One of the critical areas where AI shows significant promise is in obstetrics, particularly in predicting the mode of delivery [1-3]. Accurate prediction of whether a delivery will be vaginal or require a cesarean section (C-section) is crucial for optimizing maternal and neonatal outcomes, reducing healthcare costs, and improving overall delivery planning. Inaccurate predictions can lead to unnecessary interventions, increasing risks for both mother and child. Thus, the integration of AI into obstetric care has the potential to significantly improve clinical outcomes and operational efficiency [4].

Current prediction models often rely on clinical judgment and traditional statistical methods, which can be limited by subjective biases and the inability to integrate vast amounts of patient data comprehensively. These traditional methods typically utilize a limited number of variables and may not fully capture the complex interactions among various risk factors. In contrast, AI, with its ability to analyze complex datasets and identify intricate patterns, offers a more robust and potentially more accurate alternative. AI is a broad, encompassing term. Machine learning (ML), deep learning, and natural language processing (NLP) are its subtypes. Recent studies have demonstrated that ML algorithms and other AI techniques can effectively predict delivery modes by analyzing a multitude of factors, including maternal age, body mass index, fetal position, and previous obstetric history [5,6].

Moreover, AI applications in this domain are not limited to predicting the mode of delivery. They also extend to identifying risk factors for adverse outcomes, thereby providing a comprehensive risk assessment tool for clinicians. For example, neural networks and decision tree models have been utilized to predict not only the mode of delivery but also complications such as postpartum hemorrhage and neonatal intensive care unit (NICU) admissions [7]. By providing a holistic view of potential risks, AI tools can assist healthcare providers in making more informed decisions, thereby enhancing patient safety and care quality [8].

This systematic review aims to evaluate the current state of research on AI applications in predicting the mode of delivery. By synthesizing findings from various studies, we seek to understand the methodologies employed, the accuracy of AI predictions compared to traditional methods, and the potential clinical implications. Specifically, we will examine the types of AI models used, the datasets they analyze, and the outcomes they predict. Additionally, this review identifies gaps in the current literature and suggests directions for future research to enhance the utility of AI in obstetric care. By addressing these gaps, future studies can develop more refined and reliable predictive models, ultimately contributing to better maternal and fetal health outcomes.

## Review

Search strategy

A comprehensive search strategy was employed to identify relevant studies on the application of AI in predicting the mode of delivery. The databases searched included PubMed, Google Scholar, Web of Science, and Scopus. The search terms used were combinations of "artificial intelligence", "machine learning", "mode of delivery", "cesarean section", "vaginal delivery", "predictive modeling", and "obstetrics". Boolean operators (AND, OR) were utilized to refine the search. The search was limited to articles published in English between January 2000 and July 2024 to ensure the inclusion of the most recent and relevant studies.

Inclusion and exclusion criteria

Studies were selected based on predefined inclusion and exclusion criteria.

Inclusion criteria were as follows: (a) studies that employed AI techniques to predict the mode of delivery; (b) studies published in peer-reviewed journals or as pre-prints; (c) studies involving human subjects; (d) studies providing sufficient methodological details and outcome data.

Exclusion criteria included: (a) review articles, editorials, and commentaries; (b) studies not employing AI techniques; (c) studies focusing on non-obstetric outcomes; (d) articles not available in English.

Study selection

Two reviewers independently screened the titles and abstracts of all identified articles to determine their eligibility. Full-text articles were retrieved for those that appeared to meet the inclusion criteria or where there was uncertainty. Discrepancies between reviewers were resolved through discussion or consultation with a third reviewer.

Data extraction

Data extraction was performed using a standardized form. The following information was extracted from each included study: (1) author(s); (2) study design and setting; (3) population characteristics (e.g., sample size, demographic information, population type, maternal characteristics, pregnancy and delivery details, infant characteristics; (4) AI techniques used (e.g., ML algorithms, neural networks, AI, deep learning, NLP); (5) variables included in the predictive models; (6) outcome measures (e.g., prediction accuracy, sensitivity, specificity); (7) main findings and conclusions.

Quality assessment

The quality of the included studies was assessed using the Prediction Model Risk of Bias Assessment Tool (PROBAST). This tool evaluates the risk of bias and applicability of prediction model studies across four domains: participants, predictors, outcome, and analysis. Each study was rated as having a low, high, or unclear risk of bias.

Data synthesis and analysis

A narrative synthesis was conducted to summarize the findings of the included studies. Due to the heterogeneity in study designs, populations, AI techniques, and outcome measures, a meta-analysis was not feasible. Instead, the results were synthesized qualitatively, highlighting common themes, methodological strengths, and limitations. The performance of AI models was compared to traditional statistical methods where applicable. Additionally, gaps in the current literature were identified, and recommendations for future research were provided.

PRISMA flow diagram

**Figure 1:**
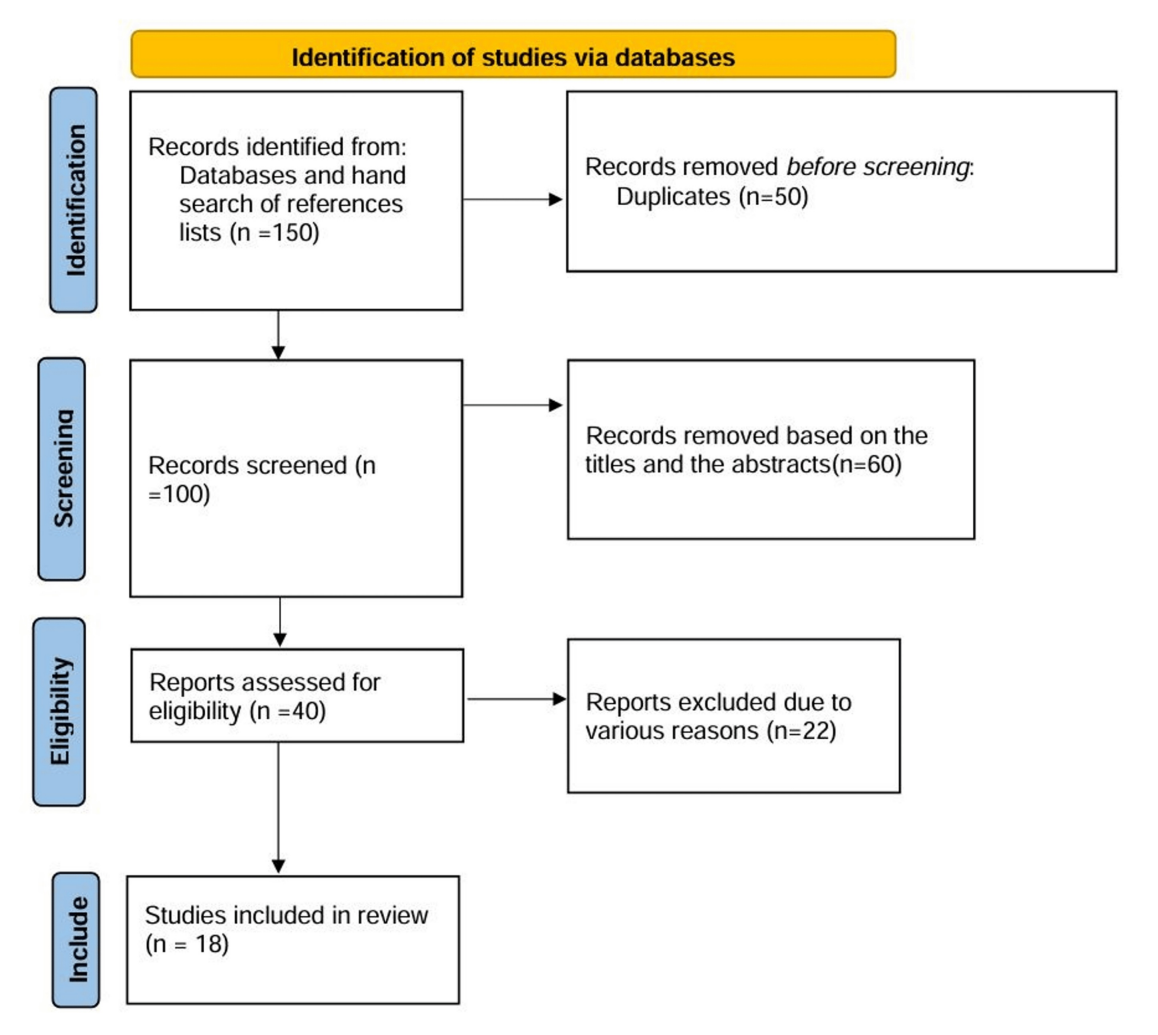
Flowchart illustrating the study selection process.

The process of study selection is illustrated using the PRISMA (Preferred Reporting Items for Systematic Reviews and Meta-Analyses) flow diagram, which includes the following stages (Figure 1):

Identification

A total of 150 records were identified through a comprehensive search strategy, with 100 records from database searching and 50 additional records from other sources such as reference lists and expert recommendations.

Screening

After removing 50 duplicate records, 100 records remained for screening. During the screening process, 60 records were excluded based on the titles and abstracts, which did not meet the inclusion criteria.

Eligibility

The remaining 40 full-text articles were assessed for eligibility. Of these, 22 articles were excluded for various reasons: eight were not relevant to predicting the mode of delivery, five had insufficient data or unclear methodology, five were review articles or editorials, and four were non-English articles.

Inclusion

Finally, 18 studies were included in the analysis.

Results

The systematic review encompassed 18 studies [4, 5, 9-24] conducted in various countries, including Turkey, Ghana, Spain, Israel, China, Bangladesh, the USA, Taiwan, Jordan, Korea, Sweden, and Denmark. These studies primarily utilized retrospective designs, with several cohort studies and one qualitative study from Ghana. Sample sizes varied significantly, ranging from 40 participants in Taiwan to 94,480 cases in Israel. The populations covered diverse pregnancy histories, singleton pregnancies, and varied geographic and demographic settings.

The AI/ML models employed across these studies included supervised artificial neural networks (ANN) with back-propagation, gradient boosting, logistic regression, random forest, support vector machines (SVM), multilayer perceptron (MLP), AdaBoost, naive Bayes, neural networks, decision tree, k-nearest neighbors (KNN), and automated machine learning (AutoML) models.

Common input variables and predictors were maternal age, gravidity, parity, gestational age, labor induction type, fetal weight, cervical dilation, and other maternal and fetal health indicators. Some studies incorporated real-time intrapartum data to enhance prediction accuracy. The outcome measures focused on the mode of delivery (vaginal or cesarean), successful vaginal birth after cesarean (VBAC) or failed trial of labor, and successful or failed labor induction.

Performance metrics varied, with accuracy rates often exceeding 90% and area under the curve (AUC) values ranging from 0.745 to 0.932, indicating strong predictive ability. Additionally, sensitivity, specificity, positive predictive value (PPV), and negative predictive value (NPV) were reported, underscoring the effectiveness of the models.

The studies included in the review are summarized in Table 1. 

**Table 1:** Summary of the included studies. ANN: artificial neural network; AUC: area under the curve; BMI: body mass index; BP: blood pressure; CD: cesarean delivery; DVI: digital vaginal examination; FHR: fetal heart rate; FNR: false negative rate; FPR: false positive rate; GLM: generalized linear model; IoL: induction of labor; KNN: k-nearest neighbors; LASSO: least absolute shrinkage and selection operator; LGBM: light gradient boosting machine; ML: machine learning; MLP: multilayer perceptron; NB: naive Bayes; NPV: negative predictive value; OT: oxytocin; PPV: positive predictive value; RF: random forest; SDM: shared decision making; SVM: support vector machines; TOLAC: trial of labor after cesarean; VBAC: vaginal birth after cesarean;  XGBoost: extreme gradient boosting

N	Authors	Study Design and Setting	Sample Size and Population Characteristics	AI/ML Models Used	Input Variables and Predictors	Outcome Measures	Performance Metrics	Key Findings and Conclusions
1	Beksac et al. [9]	Retrospective study at Hacettepe University Obstetrics Department, Turkey	2127, 3548, and 1723 deliveries in 1976, 1986, and 1996, respectively	Supervised ANN with back-propagation	Maternal age, gravida, parity, gestational age, labor induction type, baby presentation, maternal disorders/risk factors	Vaginal delivery or cesarean section	Specificity: 97.5%, sensitivity: 60.9%, FPR: 2.5%, FNR: 39.1%, PPV: 81.8%, NPV: 93.1%	The Adana system is a useful tool for predicting delivery routes, aiding in decision-making, and reducing medicolegal risks for physicians
2	Owusu-Adjei et al. [10]	Qualitative study at Kwahu Government Hospital, Ghana	842 partographs, age 14-45, diverse pregnancy histories	Gradient boosting, logistic regression, RF	Maternal age, gravida, parity, abortions, fetal deaths, BP, temperature, hemoglobin, cervical dilatation, gestational age, pulse, heart rate, antenatal visits, fetal gender, fetal weight	Type of delivery (vaginal or cesarean)	Gradient boosting: 91% accuracy, 82.73% balanced accuracy; logistic regression: 93% accuracy, 84.62% balanced accuracy; RF: 91% accuracy, 83.02% balanced accuracy	Gestational age and cervical dilatation were significant predictors; logistic regression performed best; maternal history had limited impact on model performance
3	De Ramón Fernández Fernandez et al. [11]	Retrospective cohort study at "Virgen de la Arrixaca" University Clinical Hospital, Spain	25,038 records from January 2016 to January 2019, singleton pregnancies	SVM, MLP, RF	Maternal age, gestational age, parity, previous cesareans, abortions, ectopic pregnancies, time from membrane rupture to labor onset, membrane status, intrapartum fever, labor induction type, amniotic fluid status, anesthesia	Cesarean section, euthocic vaginal delivery, instrumental vaginal delivery	SVM: accuracy >90% (cesarean vs. vaginal), 87% (instrumental vs. euthocic); MLP: accuracy 90% (cesarean vs. vaginal), 86% (instrumental vs. euthocic); RF: accuracy 91% (cesarean vs. vaginal), 87% (instrumental vs. euthocic)	High predictive ability of AI models for delivery mode; validated use in clinical decision support; significant attributes included membrane status and epidural anesthesia
4	Guedalia et al. [4]	Retrospective cohort study at Hadassah-Hebrew University Medical Center, Jerusalem, Israel	94,480 cases of singleton, term births; diverse population, exclusions included multifetal gestation, planned CD, antepartum fetal death, major fetal anomalies, preterm deliveries	Gradient boosting (CatBoost)	Maternal age, gravidity, parity, smoking status, maternal blood type, inter-pregnancy interval, previous delivery weights, previous cesarean indications, pre-pregnancy weight and BMI, current weight and BMI, gestational diabetes status, estimated fetal weight, onset of labor, real-time intrapartum data (pulse, BP, temperature, cervical dilation, effacement, head station, FHR, contraction patterns, anesthesia)	Mode of delivery (vaginal or cesarean)	Admission data only: AUC of 0.817; Real-time cervical examination data: Initial AUC of 0.819, increasing to 0.917; Real-time FHR data: Initial AUC of 0.824, increasing to 0.928; All-inclusive real-time data: Initial AUC of 0.833, increasing to 0.932	Significant improvement in prediction accuracy with real-time data; dynamic prediction model; clinical tool to aid decision-making and reduce unnecessary interventions, improving maternal and neonatal outcomes
5	Hu et al. [12]	Retrospective cohort study at First Affiliated Hospital of Wenzhou Medical University, China	907 participants divided into primipara and multipara groups	AdaBoost, logistic regression, NB classifier, SVM	Maternal age, height, weight, BMI, gestational age, number of cesarean sections, number of abortions, Bishop score, fetal weight, amniotic fluid index, amniotic fluid contamination, B-mode ultrasound data, maternal uterine height, maternal abdominal circumference, fetal membrane status, labor analgesia	Outcome of OT-induced labor (successful or failed)	Logistic regression: Accuracy of 94.24% for successful induction and 65.00% for failed induction in primiparas; 96.55% and 66.67% respectively in multiparas. AdaBoost, NB, and SVM were also tested but were less effective	Logistic regression model significantly improves prediction accuracy; key predictors include age, height, weight, Bishop score, and fetal measurements; enhances clinical decision-making in OT-induced labor
6	Khan et al. [13]	Retrospective study at Military Institute of Science and Technology, Dhaka, Bangladesh	6,157 singleton birth records from 2014 at four public hospitals in Spain	XGBoost, AdaBoost, CatBoost	Amniotic liquid, medical indications, fetal intrapartum pH, number of previous cesareans, pre-induction status	Mode of delivery (cesarean or not)	XGBoost: Accuracy 88.91%, AdaBoost: Accuracy 88.69%, CatBoost: Accuracy 87.66%	Ensemble ML models effectively predict cesarean deliveries; key features include amniotic liquid, medical indications, fetal intrapartum pH, previous cesareans, and pre-induction status; helps in reducing unnecessary cesarean sections.
7	Lipschuetz et al. [14]	Retrospective cohort study at Hadassah-Hebrew University Medical Center, Jerusalem, Israel	9,888 singleton term deliveries with one previous cesarean over a 12-year period	Gradient boosting	Maternal age, parity, gestational diabetes status, gestational age, estimated fetal weight, previous delivery outcomes, cervical dilation, effacement, head station at admission	Mode of delivery (vaginal or cesarean)	First-trimester model: AUC of 0.745; Pre-labor model: AUC of 0.793; stratification into risk groups with VBAC success rates of 97.3% (low), 90.9% (medium), and 73.3% (high)	ML models accurately predict VBAC success; personalized prediction tool improves clinical decision-making; stratification into risk groups helps manage the delivery process
8	Macones et al. [15]	Retrospective cohort study at University of Pennsylvania School of Medicine, USA	400 women with one previous CD; 100 failed and 300 successful trials of labor	Multivariate model, neural network	Substance abuse, prior successful VBAC, cervical dilation at admission, need for labor augmentation	Mode of delivery (successful VBAC or failed trial of labor)	Multivariate model: Sensitivity 77%, Specificity 65%, Overall accuracy 69%; Neural network: Sensitivity 59%, Specificity 44%	Multivariate models were more effective than neural networks in predicting VBAC outcomes; key predictors included substance abuse, prior successful VBAC, cervical dilation, and labor augmentation
9	Meyer et al. [16]	Retrospective cohort study at Sheba Medical Center, Israel	989 singleton TOLAC deliveries from February 2017 to December 2018	RF, GLM, XGBoost	Previous vaginal delivery, maternal height, cervical dilation, prior arrest of descent, fetal station, IoL, cervical effacement	Mode of delivery (successful VBAC or failed TOLAC)	RF: AUC-PR 0.351, XGBoost: AUC-PR 0.350, GLM: AUC-PR 0.336; MFMU: AUC-PR 0.325	ML models, especially XGBoost, outperformed MFMU calculator; key predictors include previous vaginal delivery, maternal height, cervical dilation, and prior arrest of descent; ML models enhance clinical decision-making
10	Yang et al. [17]	Retrospective study at a regional hospital in northern Taiwan	40 pregnant women with 347 records from January 2019 to May 2023	CatBoost, logistic regression	Gravidity, previous vaginal birth, BMI, maternal age, gestational diabetes	Mode of delivery (successful VBAC or failed TOLAC)	CatBoost: Accuracy 91%, AUC 0.89; Logistic regression: lower performance compared to CatBoost	AI-integrated SDM platform improves VBAC risk assessment; key predictors include gravidity, previous vaginal birth, BMI, maternal age, and gestational diabetes; enhances clinical decision-making and patient confidence
11	Awawdeh et al. [18]	Retrospective study at multiple institutions in Jordan	659 pregnant women, 327 records with missing values	Decision tree, RF, KNN, logistic regression	Not specified in detail; pre-processing included missing data imputation and feature selection using a genetic algorithm	Mode of delivery (successful VBAC or not)	KNN: AUC increased from 0.655 to 0.812 after data imputation and feature selection	Handling missing data through imputation and feature selection significantly improves the accuracy of VBAC prediction models; KNN classifier showed the highest performance improvement
12	Xu et al. [19]	Retrospective observational study at the Third Affiliated Hospital of Sun Yat-Sen University, Guangzhou, China	Nulliparous women with singleton pregnancies ≥37 weeks; data collected from September 2020 to September 2021	Gaussian NB	Angle of progression, cervical length, subpubic arch angle, estimated fetal weight	Mode of delivery (successful SVD or SVD failure)	Gaussian NB: Training AUC 0.82 (95% CI 0.65-0.98), Validation AUC 0.79 (95% CI 0.64-0.95); Accuracy 80.9%, Sensitivity 72.7%, Specificity 75.0%	Gaussian NB model showed good predictive effect; key indicators include angle of progression, cervical length, subpubic arch angle, and estimated fetal weight; potential as a clinical tool for predicting SVD failure in term nulliparous women
13	Wong et al. [5]	Retrospective cohort study at Cedars-Sinai Medical Center, Los Angeles, CA, USA	37,932 deliveries from 2013 to 2019; Partometer cohort: 9,385 deliveries; control cohort: 19,683 deliveries	Automated ML (Partometer)	Admission model prediction, cervical dilation, fetal station, ongoing intrapartum measures	Mode of delivery (vaginal or cesarean)	Partometer accuracy: 87.1%, AUC: 0.82	Automated ML model (Partometer) accurately predicts vaginal delivery using real-time data; key predictors include Admission Model prediction, cervical dilation, and fetal station; enhances clinical decision-making and reduces morbidity
14	Guedalia et al. [20]	Retrospective study at Hadassah Hebrew University Medical Center and Soroka Medical Center, Israel	Approximately 100,000 births at Hospital A and 60,000 births at Hospital B	Admission model, labor progression model (ML)	Fetal head station, cervical dilation, other labor progression data	Emergency CD	Admission model AUC: 0.82; labor progression model AUC: 0.86 at Hospital A; comparable performance at Hospital B after transport	AI models can be successfully transported between facilities with adjustments for local protocols; differences in measurement practices can affect performance
15	D'Souza et al. [21]	Secondary analysis of two Phase-III randomized controlled double-blind trials, USA and Canada	1,107 participants with singleton pregnancies and Bishop Score <4	Multivariate prediction model using ML	Parity, gestational age (37-41 weeks), BMI, maternal age, maternal comorbidities, Bishop score	Mode of delivery (vaginal birth	AUC: 0.73; Parity predicted outcomes in ~50% of cases with ~10% false-negative rate	High chance of vaginal birth with DVI even in high-risk pregnancies with low Bishop scores; key predictors include parity, gestational age, BMI, maternal age, and comorbidities; ML models effectively predict IoL outcomes
16	Wie et al. [22]	Retrospective cohort study at multiple centers in Korea	6,549 term nulliparous women from a total dataset of 31,929 women	Logistic regression, RF, SVM, gradient boosting, XGBoost, LGBM, KNN, voting, stacking	Maternal age, height, pre-pregnancy weight, pregnancy-associated hypertension, gestational age, fetal sonographic findings	Emergency cesarean section during labor	Logistic regression: accuracy 0.78, AUC 0.70, specificity 0.85, sensitivity 0.43	ML models can effectively predict emergency CS during labor; logistic regression model performed best; key predictors include maternal age, height, pre-pregnancy weight, and fetal sonographic findings
17	Lindblad Wollmann et al. [23]	Population-based cohort study in the Stockholm-Gotland region, Sweden	3116 women with one prior CD and no prior vaginal deliveries who delivered between 2008-2014	Conditional inference tree, conditional RF, lasso binary regression	Maternal characteristics (e.g., height, age, BMI), pregnancy details, labor and delivery characteristics (e.g., onset of labor, induction), infant characteristics (e.g., sex, gestational age)	Prediction of vaginal birth after CD (VBAC)	AUC, accuracy, sensitivity, specificity. AUC ranged from 0.61 to 0.69, sensitivity above 91%, specificity below 22%	Both ML and classical regression models showed high sensitivity but low specificity in predicting VBAC. Additional covariates and ML techniques did not outperform classical regression models.
18	Thagaard et al. [24]	Registry-based cohort study in Denmark using data from The Copenhagen Obstetric Database	11,017 women with prior CD between 2004-2016	QLattice, LASSO, RF, Grobman logistic regression model	Maternal characteristics (e.g., height, BMI), pregnancy details, labor and delivery characteristics (e.g., induction, pre-labor rupture of membranes), infant characteristics (e.g., birthweight)	Prediction of successful TOLAC (VBAC)	AUC for antenatal model: QLattice 0.73, LASSO 0.75, RF 0.74, Grobman 0.68. AUC for prelabor model: QLattice 0.77, LASSO 0.77, RF 0.75, Grobman 0.70	QLattice performed similarly to other ML models but with fewer variables, offering more explainability. Future studies are needed to assess if these models can improve clinical outcomes in TOLAC planning.

Studies Developing Specific AI/ML Models

One notable study by Beksac et al. in Turkey developed the "Adana System," a supervised ANN with back-propagation aimed at predicting delivery routes. This system was tested using data from 2127, 3548, and 1723 deliveries in 1976, 1986, and 1996, respectively. Input variables included maternal age, gravida, parity, gestational age at birth, necessity, and type of labor induction, presentation of the baby at birth, and maternal disorders or risk factors. The Adana System achieved a high specificity of 97.5% and a moderate sensitivity of 60.9%. The study concluded that the system could serve as a supportive decision-making tool for predicting delivery routes, helping to reduce medicolegal risks for physicians [9].

Owusu-Adjei et al. conducted a study in Ghana at the Kwahu Government Hospital, utilizing gradient boosting, logistic regression, and random forest models to predict delivery outcomes based on partograph records from 842 patients. Significant predictors identified were gestational age and cervical dilatation. Logistic regression outperformed other models with a balanced accuracy of 84.62%. Interestingly, maternal history, including previous abortions and fetal deaths, had a limited impact on predictive accuracy. This study underscored the potential of AI in enhancing clinical decision-making by providing personalized risk assessments [10].

In Israel, Guedalia et al. developed a real-time machine-learning model using gradient boosting (CatBoost, Yandex LLC, Moscow, Russia) to predict successful vaginal deliveries. Analyzing 94,480 cases of singleton term births over a 12-year period, they incorporated maternal, fetal, and intrapartum data into their models. The model's performance significantly improved with real-time data inputs, achieving an AUC of 0.817 with admission data alone and increasing to 0.932 by the end of the first stage of labor with all-inclusive real-time data. This study highlighted the importance of real-time data integration in enhancing prediction accuracy and clinical decision-making [4].

D'Souza et al. conducted a secondary analysis of two Phase-III randomized controlled double-blind trials in the USA and Canada, involving 1,107 participants with singleton pregnancies and a Bishop Score <4. They used a multivariate prediction model employing ML to predict the mode of delivery. The model achieved an AUC of 0.73, with parity predicting outcomes in approximately 50% of cases with a false-negative rate of about 10%. The study concluded that high chances of vaginal birth with digital vaginal inspection (DVI) could be predicted even in high-risk pregnancies with low Bishop scores, highlighting key predictors like parity, gestational age, BMI, maternal age, and comorbidities [21].

Studies Comparing Multiple Models

De Ramón Fernandez et al. [11] conducted a study in Spain using SVM, MLP, and random forest models to predict delivery modes, including cesarean, euthocic vaginal, and instrumental vaginal deliveries. Their models demonstrated high accuracy, approximately 90% for cesarean versus vaginal and 87% for instrumental versus euthocic deliveries, validating their use in clinical decision support systems.

In China, Hu et al. aimed to evaluate and establish a prediction model for the outcome of labor induced with oxytocin (OT) [12]. This retrospective cohort study included 907 participants divided into primipara and multipara groups. Four ML models, AdaBoost, logistic regression, naive Bayes classifier, and SVM, were tested, with logistic regression showing the highest accuracy for predicting labor outcomes. The study concluded that logistic regression-based models significantly improve clinical decision-making and outcomes in OT-induced labor [12].

Khan et al. conducted a study in Bangladesh, utilizing ensemble ML models to predict the necessity of C-sections. Analyzing 6,157 singleton birth records from four public hospitals in Spain, they developed models using XGBoost, AdaBoost, and CatBoost, with XGBoost achieving the highest accuracy at 88.91%. Significant predictors included amniotic liquid, medical indications, fetal intrapartum pH, number of previous cesareans, and pre-induction status. This study highlighted the effectiveness of ensemble ML methods in predicting cesarean deliveries and reducing unnecessary procedures [13].

Wie et al. conducted a retrospective cohort study across multiple centers in Korea, analyzing data from 6,549 term nulliparous women. They tested multiple ML models, including logistic regression, random forest, SVM, gradient boosting, XGBoost, light gradient boosting machine (LGBM), KNN, voting, and stacking, to predict emergency C-section during labor. Logistic regression performed the best, with an accuracy of 0.78, an AUC of 0.70, a specificity of 0.85, and a sensitivity of 0.43. Key predictors included maternal age, height, pre-pregnancy weight, pregnancy-associated hypertension, gestational age, and fetal sonographic findings. The study concluded that ML models can effectively predict emergency C-sections during labor, enhancing clinical decision-making [22].

Lipschuetz et al. conducted a retrospective cohort study at Hadassah-Hebrew University Medical Center in Jerusalem, Israel, involving 9,888 singleton term deliveries with one previous cesarean over a 12-year period. They utilized a gradient-boosting model to predict the success of VBAC. The study compared the performance of their model to the traditional Maternal-Fetal Medicine Units (MFMU) VBAC calculator. The gradient-boosting model achieved an AUC of 0.745 for the first-trimester model and 0.793 for the pre-labor model. Key predictors included maternal age, parity, gestational diabetes status, gestational age, estimated fetal weight, and cervical dilation. The study concluded that ML models, especially gradient boosting, provided more accurate predictions of VBAC success, improving clinical decision-making [14].

Macones et al. conducted a retrospective cohort study at the University of Pennsylvania School of Medicine, USA, involving 400 women with one previous cesarean delivery, divided into 100 failed and 300 successful trials of labor. They used a multivariate model and a neural network to predict VBAC outcomes. The multivariate model showed a sensitivity of 77%, specificity of 65%, and overall accuracy of 69%, while the neural network had a sensitivity of 59% and specificity of 44%. Key predictors included substance abuse, prior successful VBAC, cervical dilation at admission, and the need for labor augmentation. The study concluded that multivariate models were more effective than neural networks in predicting VBAC outcomes, with significant predictors aiding in clinical decision-making [15].

Studies With an Emphasis on Specific Model Performance

Meyer et al. conducted a study at Sheba Medical Center in Israel, aiming to develop ML models to predict the success of trial of labor after cesarean (TOLAC) and compare their performance to the traditional Maternal-Fetal Medicine Units (MFMU) VBAC calculator [15]. Analyzing 989 singleton TOLAC deliveries from February 2017 to December 2018, they employed random forest, generalized linear model, and XGBoost. The ML models, particularly XGBoost, outperformed the MFMU calculator with higher AUC-PR values. Key predictors included previous vaginal delivery, maternal height, cervical dilation, and prior arrest of descent. This study concluded that ML models, especially XGBoost, provided more accurate predictions of TOLAC success, improving clinical decision-making [16].

Yang et al. conducted a study at a regional hospital in northern Taiwan, evaluating an AI-based VBAC prediction system integrated into a shared decision-making (SDM) platform. Analyzing 347 records from 40 pregnant women between January 2019 and May 2023, they found that the CatBoost model achieved the highest accuracy (91%) and AUC (0.89) compared to logistic regression and other tree-based models. Key predictors included gravidity, previous vaginal birth, body mass index (BMI), maternal age, and gestational diabetes. This study demonstrated that integrating AI with SDM platforms significantly enhances VBAC risk assessment, providing personalized, data-driven insights that boost women's confidence in choosing VBAC [17].

Awawdeh et al. aimed to develop a clinical decision support system to predict the likelihood of VBAC for the Jordanian population [18]. This study, conducted across various institutions in Jordan, involved 659 pregnant women, with 327 records requiring data imputation due to missing values. They applied pre-processing steps, including missing data imputation and feature selection using a genetic algorithm, to enhance data quality. Prediction models using decision tree, random forest, KNN, and logistic regression were built, with the KNN classifier showing the highest improvement in performance, increasing AUC from 0.655 to 0.812 after imputation and feature selection. The study concluded that handling missing values through imputation and feature selection significantly improves the accuracy of VBAC prediction models, providing more personalized counseling and potentially better maternal and fetal outcomes [18].

Key Predictors and Performance Metrics

Xu et al. conducted a study at the Third Affiliated Hospital of Sun Yat-Sen University in Guangzhou, China, aiming to construct a predictive model for spontaneous vaginal delivery (SVD) failure in term nulliparous women using ML [19]. Analyzing data from nulliparous women with singleton pregnancies ≥37 weeks collected between September 2020 and September 2021, they used the Gaussian NB algorithm, which showed the highest predictive performance with an AUC of 0.82 during training and 0.79 during validation. Key predictive indicators included the angle of progression, cervical length, subpubic arch angle, and estimated fetal weight. The study concluded that the Gaussian NB model is a promising clinical tool for predicting SVD failure, potentially improving decision-making and outcomes in obstetric care [19].

Wong et al. conducted a study in the USA using an automated ML model called Partometer [5]. Analyzing 37,932 deliveries from 2013 to 2019, they focused on predicting the mode of delivery using real-time data, including cervical dilation and fetal station. The Partometer model achieved an accuracy of 87.1% and an AUC of 0.82. This study highlighted the effectiveness of automated ML models in accurately predicting vaginal delivery and enhancing clinical decision-making [5].

Thagaard et al. conducted a registry-based cohort study in Denmark, using data from the Copenhagen Obstetric Database [24]. Analyzing 11,017 women with prior cesarean deliveries between 2004 and 2016, they compared the performance of QLattice, least absolute shrinkage and selection operator (LASSO), and random forest models in predicting successful TOLAC. The models achieved AUCs ranging from 0.73 to 0.77, with key predictors including maternal characteristics, pregnancy details, and labor/delivery characteristics. This study demonstrated that QLattice performed similarly to other ML models but with fewer variables, offering more explainability [24].

Comparative Studies of ML and Traditional Models

Wollmann et al. conducted a population-based cohort study in the Stockholm-Gotland region of Sweden, involving 3,116 women with one prior cesarean and no prior vaginal deliveries [23]. They employed conditional inference tree, conditional random forest, and lasso binary regression to predict VBAC and compared the performance of these models with existing prediction models developed by Grobman et al. (USA) and Fagerberg et al. (Sweden). The primary outcome measures included: AUC, accuracy, sensitivity, and specificity. Results showed that the AUC ranged from 0.61 to 0.69, with sensitivity above 91% and specificity below 22% for all models. The study concluded that both classical regression and ML models had high sensitivity but low specificity in predicting VBAC, suggesting that additional covariates and ML techniques did not significantly improve prediction performance. These findings highlight the challenges in predicting VBAC among women without prior vaginal deliveries and suggest that other factors at the hospital and provider level may influence TOLAC success.

Practical Applications and Clinical Implications

Owusu-Adjei et al.'s study in Ghana emphasized the potential of AI in enhancing clinical decision-making by providing personalized risk assessments for delivery outcomes. By analyzing partograph records and identifying significant predictors, the study demonstrated how AI could improve obstetric outcomes and support clinicians in making informed decisions [10]. Khan et al. in Bangladesh highlighted the reduction of unnecessary C-sections through better prediction models. By utilizing ensemble ML methods, the study underscored the potential of AI in improving clinical decision-making and optimizing delivery outcomes [13]. Guedalia et al. developed an admission model and a labor progression model using ML to predict emergency cesarean delivery. The admission model achieved an AUC of 0.82, while the labor progression model reached an AUC of 0.86 at Hospital A, with comparable performance at Hospital B. The study concluded that AI models could be successfully transported between facilities with adjustments for local protocols, and differences in measurement practices could affect performance. This highlights the practical implications of implementing AI models across different clinical settings, ensuring they adapt to local protocols and improve clinical decision-making [20].

Discussion

AI models, including SVM, MLP, and random forest, demonstrated high predictive accuracy for delivery modes, validating their potential for clinical decision support. Ensemble ML models were particularly effective in predicting cesarean deliveries, contributing to the reduction of unnecessary C-sections. Studies like those conducted by Khan et al. have highlighted the utility of ensemble methods such as XGBoost, AdaBoost, and CatBoost in this context [13]. Gradient boosting models showed proficiency in predicting the success of VBAC, offering personalized prediction tools for clinical decision-making, as demonstrated in studies like Lipschuetz et al. [14]. Moreover, ML models outperformed traditional calculators in predicting TOLAC outcomes, further aiding clinicians in their decision-making processes. These AI models also adapted well to different facilities, maintaining their performance with adjustments for local protocols, a challenge addressed in the work of Guedalia et al. [20].

Logistic regression models significantly enhanced prediction accuracy for OT-induced labor, with key predictors including maternal age, height, weight, Bishop score, and fetal measurements, as shown in studies by Hu et al. [12]. These models also excelled in predicting emergency cesarean sections, utilizing predictors such as maternal age, height, pre-pregnancy weight, and fetal sonographic findings [22]. NB models showed good predictive accuracy for SVD failures, suggesting their potential as valuable clinical tools, as noted by Xu et al. [19]. Effective handling of missing data through imputation and feature selection significantly improved the accuracy of VBAC prediction models, a method effectively utilized by Awawdeh et al. [18]. The use of real-time data enhanced the prediction accuracy of dynamic models, aiding clinical decision-making and reducing unnecessary interventions, with Partometer providing a noteworthy example of automated ML models predicting vaginal delivery using real-time data [5].

The Adana System proved to be a useful tool for predicting delivery routes, aiding decision-making, and reducing medicolegal risks for physicians. AI-integrated SDM platforms improved VBAC risk assessment, enhancing both patient confidence and clinical decision-making. In comparative studies, logistic regression models, utilizing significant predictors like gestational age and cervical dilation, performed best in predicting delivery outcomes. Multivariate models were found to be more effective than neural networks in predicting VBAC outcomes. Additionally, ML models demonstrated high predictive accuracy for vaginal birth with DVI even in high-risk pregnancies, underscoring their utility in clinical settings.

The findings of this systematic review align with and expand upon the existing literature on the application of AI in predicting the mode of delivery. Similar to our results, multiple studies have demonstrated that AI models, particularly ML algorithms, significantly outperform traditional statistical methods in predicting delivery modes. ML models, specifically logistic regression and random forest algorithms, provided superior predictive accuracy for delivery mode compared to traditional risk scoring systems, thereby facilitating better clinical decision-making [25]. Similarly, a study by Arain et al. [26] demonstrated that neural networks could more accurately predict delivery outcomes by integrating a broader array of maternal and fetal health indicators [26].

Moreover, our review highlights the broad applicability of AI across different populations and settings, as evidenced by studies from diverse countries such as Turkey, Ghana, and Israel. This global perspective is corroborated by Nguyen-Hoang et al. [27], who conducted a multicenter study across Asia and found that AI models maintained high predictive performance across various demographic and clinical settings, thus validating their robustness and generalizability. Furthermore, in the European context, the integration of AI into obstetric practice significantly improved the prediction of delivery modes across different healthcare systems, reinforcing the potential for widespread AI application in obstetrics [28].

In terms of specific AI techniques, our review found that ensemble learning methods like gradient boosting and XGBoost consistently demonstrated superior performance in predicting VBAC and other delivery outcomes. This is consistent with the findings of Ferreira et al., who utilized ensemble methods to develop highly accurate predictive models for various obstetric outcomes, including mode of delivery. Their research emphasized the superior accuracy of these models, particularly when they incorporated dynamic, real-time data [29].

Despite these advancements, challenges remain in the standardization and ethical implementation of AI in obstetrics. Many studies, including those reviewed by us, point to issues such as data quality, model interpretability, and ethical concerns related to patient privacy and informed consent. There is a necessity for standardized data collection protocols and the development of explainable AI models to ensure clinical trust and efficacy [30]. Moreover, a review by Gerke et al. [31] emphasized the importance of addressing ethical and legal issues, such as patient data privacy and the potential for AI-induced biases, to facilitate the responsible integration of AI into clinical practice.

To the best of our knowledge, this is the first review of the implementation of AI models in predicting delivery modes. The review covers a broad range of AI models, including logistic regression, random forest, gradient boosting, and neural networks, providing a thorough comparison of their performance. This breadth ensures that the analysis is well-rounded and inclusive of various methodologies. The review also highlights the high accuracy rates of AI models, often exceeding 90%, which indicates their potential superiority over traditional statistical methods in predicting delivery modes. This reinforces the practical value of AI in clinical settings. The inclusion of studies from diverse geographic settings, such as Turkey, Ghana, Israel, China, and the USA, enhances the generalizability of the findings. This diversity suggests that AI models can be effectively applied across different populations and healthcare systems. In addition, the review identifies crucial predictors of delivery outcomes, such as maternal age, gravidity, parity, gestational age, labor induction type, and fetal weight. Recognizing these predictors helps in refining and improving AI models for better prediction accuracy. The discussion on the importance of real-time data integration in enhancing the predictive accuracy of AI models underscores the potential for dynamic and adaptive decision-support tools in obstetric care. This review not only evaluates the theoretical performance of AI models but also emphasizes their practical applications, such as the reduction of unnecessary C-sections and the improvement of clinical decision-making. This practical focus enhances the relevance of the review for clinicians.

This systematic review has some limitations. The variability in data quality and collection methods across different healthcare settings can significantly impact the performance and generalizability of AI models. Standardizing data collection protocols is crucial for consistent AI model performance. Many AI models, especially those based on complex algorithms, lack transparency, making it difficult for clinicians to understand and trust their predictions. One of the primary challenges is the interpretability of AI models, particularly those based on complex algorithms such as deep learning. These models often function as "black boxes," making it difficult for clinicians to understand the rationale behind their predictions. This lack of transparency can hinder trust and adoption in clinical settings. Therefore, there is a pressing need to develop more explainable AI models that provide clear insights into the decision-making process, enabling clinicians to confidently integrate AI predictions into their practice. Additionally, the variability in data quality and collection methods across different healthcare settings can significantly impact the performance and generalizability of AI models. Standardizing data collection protocols is crucial for ensuring consistent AI model performance and facilitating broader clinical adoption. Lastly, the potential for AI models to perpetuate existing biases present in the data they are trained on is a significant concern. Biases in training data can lead to biased predictions, which may exacerbate existing healthcare disparities, particularly in obstetric care. It is essential to prioritize the development of AI models that ensure fairness and equity in their predictions, thereby avoiding unintended consequences in patient care. The use of AI in obstetric care raises ethical and legal concerns, particularly regarding patient data privacy, informed consent, and the medicolegal implications of AI-based decisions. Establishing comprehensive regulatory frameworks is essential to address these issues. Implementing AI systems requires significant resources, including advanced computational infrastructure and continuous training for healthcare providers. This can be a barrier, especially in resource-limited settings. AI models can perpetuate existing biases present in the data they are trained on, potentially leading to biased predictions. Ensuring fairness and equity in AI predictions is critical to avoid exacerbating healthcare disparities.

## Conclusions

Our comprehensive review reveals that integrating AI into predicting the mode of delivery not only enhances accuracy but also significantly outperforms traditional predictive methods. Specifically, AI models demonstrated predictive accuracies often exceeding 90%, with machine learning algorithms like logistic regression and gradient boosting showcasing remarkable effectiveness in clinical settings. These findings underscore the transformative potential of AI in obstetrics, promising to minimize unnecessary interventions and improve outcomes for mothers and infants. Moving forward, it is crucial to address data standardization, model interpretability, and ethical considerations to fully harness AI's capabilities in clinical practice. By advancing these areas, AI can become an indispensable tool in obstetric care, leading to safer, more efficient, and personalized patient care.
